# Physiotherapists’ User Acceptance of a Lower Limb Robotic Exoskeleton in Specialized Rehabilitation: Qualitative Exploratory Study

**DOI:** 10.2196/68233

**Published:** 2025-04-16

**Authors:** Anstein Olimb Hillkirk, Kirsti Skavberg Roaldsen, Hege Mari Johnsen

**Affiliations:** 1 Department of Health and Nursing Science, Faculty of Health and Sport Sciences University of Agder Grimstad Norway; 2 Center for Research and Innovation Sunnaas Rehabilitation Hospital Nesodden Norway; 3 Department of Neurobiology, Care Sciences and Society Karolinska Institutet Stockholm Sweden; 4 Department of Health and Care Sciences, Faculty of Health Sciences UiT The Arctic University of Norway Tromsø Norway

**Keywords:** assistive technology, clinical implementation, deductive analysis, robot-assisted gait training therapy, RAGT, user experiences, unified theory of acceptance and use of technology, UTAUT, rehabilitation

## Abstract

**Background:**

Robotic lower limb exoskeletons have emerged as promising tools in the clinical rehabilitation of patients with lower limb paralysis due to neurological disease, stroke, or spinal cord injury. Identified benefits in gait function rehabilitation include improved gait function, cardiovascular effects, enhanced training quality, patient motivation, and reduced physical and psychological workload for therapists. Despite the identified benefits, the successful adoption of this technology largely depends on therapists’ user acceptance.

**Objective:**

This study aims to explore physiotherapists’ perceptions of using robot-assisted lower-limb gait training in specialized neurological rehabilitation using the unified theory of acceptance and use of technology framework.

**Methods:**

A qualitative, exploratory research design with a deductive approach was used. Semistructured interviews were conducted with 7 expert physiotherapists in a Norwegian specialized rehabilitation hospital. Data collection and analysis were guided by the unified theory of acceptance and use of technology framework.

**Results:**

The physiotherapists’ use of lower limb exoskeletons was greatly influenced by perceived benefits for patients or challenges, such as usability issues, the time required for adjustment to each patient, and the lack of personnel resources to facilitate their use. Thus, perceived usefulness and facilitating conditions (or lack thereof) had a great influence on the physiotherapists’ intentions to use and the actual use of the exoskeleton.

**Conclusions:**

This study identified several factors influencing the physiotherapists’ acceptance and integration of the lower limb exoskeleton. Available resources, such as time and personnel, were emphasized as important factors to increase the use of the exoskeleton in specialized rehabilitation. Our findings may inform service providers and engineers in specialized neurological rehabilitation settings.

## Introduction

### Background

The use of robot-assisted gait training (RAGT) for patients with lower-limb paralysis due to neurological disease, stroke, or spinal cord injury has seen a global increase over the past 15 years [1-3]. The appeal of this technology in gait function rehabilitation is manifold, including improved gait function, cardiovascular benefits, enhanced training quality, objective measurements, patient motivation, and reduced physical and psychological workload for therapists [2,4-6].

Traditionally, treatment aimed at improving gait function requires frequent and meticulous follow-up several times a day by at least 1 physiotherapist to have a significant impact [7,8], which imposes significant resource burdens. To mitigate these challenges, lower limb exoskeletons have emerged as promising tools in clinical rehabilitation to allow intensive, high repetition of the gait cycle in individuals with locomotor disability, with reduced therapist effort [9]. Among health care professionals who interact directly with this technology, physiotherapists are expected to play a crucial role due to their specialized expertise. Their experiences and perceptions are likely to significantly impact the integration of exoskeletons into clinical practice because of both their academic competencies and hands-on experience in the rehabilitation of gait function. Physiotherapists are also proficient in the application of different modalities and new technology in clinical practice. Therefore, physiotherapists’ perspectives are likely to be fundamental in the successful implementation of this technology.

The literature provides valuable insights into the experiences of physiotherapists regarding benefits, disadvantages, and practical considerations with RAGT [2,3,10-14]. Studies have explored physiotherapists’ experiences with a diverse array of robotic exoskeletons in different treatment settings. Most studies have revealed a complex interplay of benefits and disadvantages. For example, using RAGT compared to traditional rehabilitation, physiotherapists have observed similar benefits to those experienced by patients, including increased training volume within a single session, improved gait, and better balance [2,12]. They also report that benefits include reduced physical strain and fatigue, improved gait patterns, the ability to stand upright, experiencing greater control and safety, and expansion of where rehabilitation can take place [2,10]. Studies have also reported challenges related to the use of robotic exoskeletons, such as the time required to calibrate the equipment, the time needed for training in its use, user-friendliness, adaptability to patients with various physical adaptations, and ensuring patient comfort and safety [3,10,13]. Physiotherapists have also expressed concerns about the technology’s clinical integration, addressing resource requirements, overcoming equipment-specific challenges, and managing expectations of the technology [10,11,14].

While physiotherapists remain optimistic about the technology’s ongoing evolution, the existing literature lacks a focus on the end user’s perspective. Studies stress that this viewpoint is vital in the development and deployment of robot-assisted training tools [3]. They also assert that the successful implementation of these tools largely depends on therapists’ perceived usefulness and user acceptance [15,16].

Knowing that the successful adoption of robot assistive technology largely depends on therapists’ user acceptance, there are still gaps in fully comprehending the nuances of physiotherapists’ perspectives. Therefore, this study aims to explore physiotherapists’ perceptions of using robot-assisted lower-limb gait training in specialized neurological rehabilitation, using the unified theory of acceptance and use of technology (UTAUT) framework [17]. UTAUT is among the most used frameworks in explaining which determinants affect the acceptance of various health care technologies through different user groups, settings, and countries [18]. UTAUT has also proven to be valid for assessing technology acceptance among therapists in rehabilitation [19,20]. We foresee that the findings could potentially inform exoskeleton engineering, clinicians, researchers, and service providers while also contributing to the body of knowledge on RAGT in specialized neurological rehabilitation settings in Norway and beyond.

### The Rehabilitative Robotic Exoskeleton

**Figure 1:**
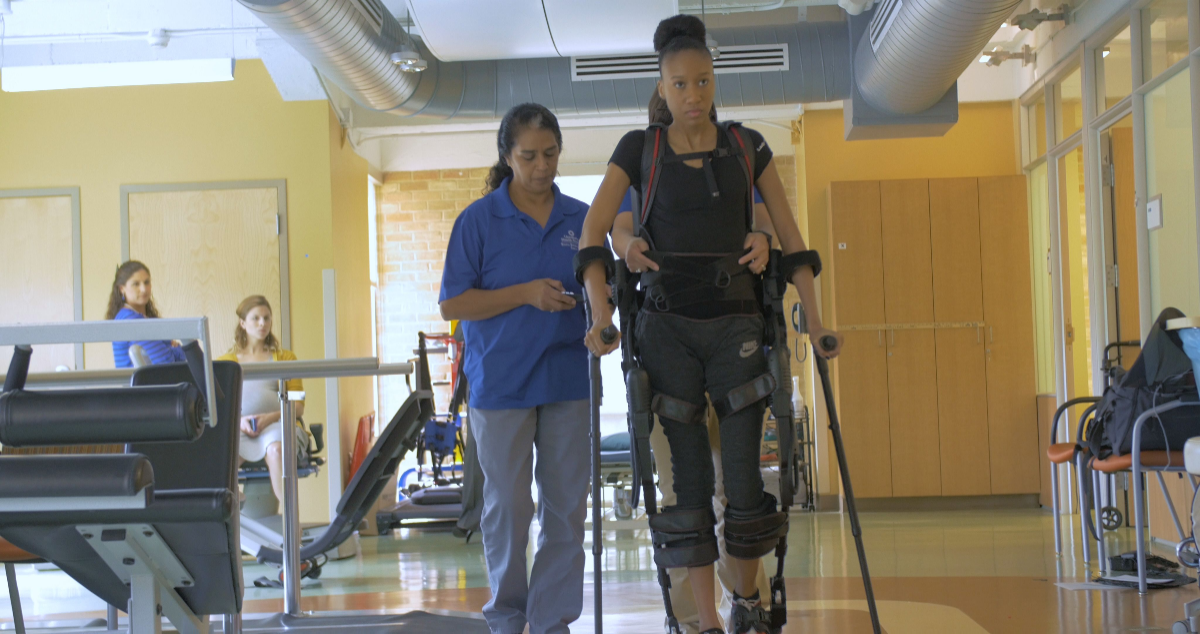
Overground exoskeleton, Ekso GT (Ekso Bionics Holdings Inc).

Robotic technology for gait training can be divided into 2 main categories: end effector and exoskeleton, with lower limb robotic exoskeletons further classified as “static” or “overground,” where movement around the environment is possible [1]. This paper focuses on overground exoskeletons, which are of 2 main types: assistive and rehabilitative [21]. Assistive robotic exoskeletons aim to serve as aids in daily life, while rehabilitative robotic exoskeletons aim to restore gait function in patients, particularly those with neurological conditions. There are several manufacturers of this type, but this paper refers to the Ekso GT model (Ekso Bionics Holdings Inc) [5], which is shown in Figure 1.

Gait rehabilitation robots commonly feature an external motorized exoskeleton that follows the legs on both sides. Movements are triggered by the physiotherapist with hand control or by the patient herself or himself using leg movement or upper body momentum. An actuator in the knee and hip joint then initiates movement. Gait robots require support from the upper body or a therapist to maintain balance [1]. Furthermore, we will refer to the Ekso GT by the term “exoskeleton” in this paper.

## Methods

### Study Design and Method

A qualitative exploratory research design with a deductive approach was used along with individual semistructured interviews [22].

### Participants and Recruitment

A purposive sampling of physiotherapists was undertaken at a Norwegian specialized rehabilitation hospital based on the following inclusion criteria: physiotherapists with clinical experience using the exoskeleton, holding a minimum of 50% position, and having ≥2 years of experience in traditional gait rehabilitation. The head of the technological intervention center at the hospital assisted in disseminating invitations to physiotherapists who met the inclusion criteria. Initially, all 9 eligible physiotherapists responded with interest and were given a study information letter emphasizing confidentiality and voluntary participation. A total of 2 of the recruited participants chose to withdraw before the interview. Thus, our final sample consisted of 7 physiotherapists.

All included informants worked at Norway’s largest and most advanced rehabilitation hospital for individuals who have experienced severe illness or injury. The institution undertakes nationwide tasks and responsibilities and functions as a university-affiliated institution. The hospital is accredited according to the international standards of the Commission on Accreditation of Rehabilitation Facilities.

### Data Collection

**Figure 2:**
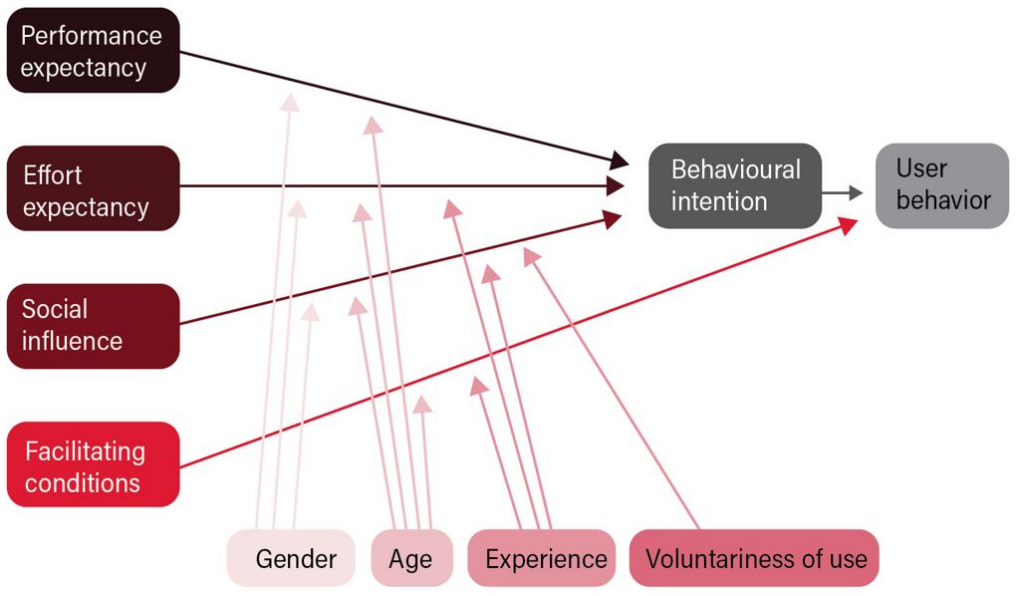
The unified theory of acceptance and use of technology framework.

Individual interviews were chosen as they are suitable for capturing diverse experiences and subjective perceptions while preserving unbiased responses from the informants [22]. A semistructured interview guide was developed by the first author (AOH) and was based on the UTAUT framework (Figure 2 [17]).

UTAUT posits 3 determinants directly influencing behavioral intention (performance expectancy, effort expectancy, and social influence) and 2 determinants directly impacting use behavior (facilitating conditions and behavioral intention). Together, these 5 determinants, along with use behavior itself, comprise the 6 main constructs of user acceptance according to the UTAUT. In addition, the framework includes 4 moderators (gender, age, technology experience, and voluntariness of use). Table 1 shows how the main constructs of the UTAUT framework were applied in the development of the interview guide.

**Table 1:** Interview questions guided by the unified theory of acceptance and use of technology (UTAUT) framework.

Main constructs of the UTAUT framework	Interview questions
Performance expectancy	How do you perceive the usefulness of the exoskeleton in treatment? How does its usefulness compare to other or traditional gait training methods?
Effort expectancy	How do you experience the user-friendliness of the exoskeleton? What do you think are important factors for the technology to be perceived as more user-friendly in gait training?
Social influence	In what ways are your use of the exoskeleton influenced by colleagues’ or the management’s attitude towards the exoskeleton? In what way do you feel that having knowledge and skills in using this technology provides any benefits for you as an employee?
Facilitating conditions	How is use of the exoskeleton organized in the department? (time, resources, etc) What type of training have you received in using the exoskeleton?
Use behavior	How often do you use the exoskeleton? Do you choose the exoskeleton over other treatment methods?
Behavioral intention	What are your intentions for future use of the exoskeleton? What possibly promotes or hinders your use of the exoskeleton?

The interview guide was pilot-tested by a physiotherapy researcher at the specialized rehabilitation hospital who was familiar with RAGT and qualitative research. The pilot testing led to changes to a couple of questions; one of the questions proved to be leading, and the other was a yes or no type of question.

In the semistructured interview setting, the open-ended questions were based on the main constructs of the UTAUT framework (Table 1). These questions were followed up with probing questions to further explore responses and the topic of interest [22]. The first author (AOH) conducted all the interviews. Due to the COVID-19 pandemic and physical distancing requirements, all the interviews were conducted digitally using Microsoft Teams (version 1.5.00.9163) or Zoom (version 5.10.1; Zoom Communications Inc). The interviews lasted from 35 to 70 minutes, with a mean of 60 minutes. The interviews were audio recorded for transcription purposes using a digital recorder (web form) with approval from the data protection officer at the University of Agder.

### Data Analysis

The first author (AOH) transcribed all the interviews and conducted the initial analysis combining deductive and inductive approaches [23]. First, the transcripts were read through to identify units of meaning that were relevant to the research question. Second, the units of meaning were placed under appropriate main categories aligned with the UTAUT constructs in an analysis matrix, representing the deductive part of the content analysis. Five of the 6 main constructs of the UTAUT were viewed as predetermined main categories and served as the deductive framework as follows: performance expectancy, effort expectancy, social influence, facilitating conditions, and behavioral intention (Table 1). Data concerning use behavior were not included in the content analysis but were considered demographic data regarding the actual use of the exoskeleton. Thus, the deductive analysis was used based on 5 of the 6 predetermined main categories of the UTAUT framework.

Next, all meaning units sorted under each category were reread inductively to identify possible subcategories. Furthermore, the units of meaning were condensed, coded, abstracted, and grouped into subcategories. The final part of the analysis process, as described in Table 2, represented the inductive part of the content analysis, and it allowed for the emergence of unanticipated subcategories. The first author (AOH) discussed all the findings of the deductive and inductive analyses with the last author (HMJ) until a consensus was achieved. Furthermore, the identified subcategories were discussed, and a consensus was reached among the 3 authors. The analysis was partly conducted with NVivo Pro 12 software (Lumivero).

In alignment with reporting qualitative research, the prevalence of participants’ responses is generally presented using descriptors, such as “most,” “many,” and other equivalent terms. The manuscript preparation adhered to the COREQ (Consolidated Criteria for Reporting Qualitative Research) checklist.

**Table 2:** Example of the inductive part of the content analysis.

Main category	Subcategory	Code	Meaning unit (participant statement)
Performance expectancy	Advantages of using the exoskeleton	The exoskeleton contributes to controlled and focused working conditions.	“What can be nice is that it’s [the exoskeleton] slow and you can correct and have more focus on quality in gait training. I worked with stroke patients who don’t use the affected side much without the robot. When using the robot, you can work on focusing on not letting them compensate with a shorter stance phase on the affected side.”

### Ethical Considerations

Ethics approval for this study was granted from the Norwegian Agency for Shared Services in Education and Research (578944) and the ethics committee at the faculty of health and sport sciences at the University of Agder. All participants received written and oral information about the project, and written informed consent was secured from all participants via email before the scheduled interviews. The first author (AOH), who conducted the interviews, had not physically met the participants before the actual interviews. The participants did not receive any compensation for their contribution in the study.

To secure confidentiality, study IDs were assigned to each participant that replaced their names in transcripts. Names and participant numbers were stored separately in a secure location. In addition, direct personally identifiable data, such as age, gender, and experience, were not directly reported in this paper but summarized under demographic data in the Results section because all the participants resided in the same unit at an identified hospital. Furthermore, participant numbers were not displayed in presenting direct quotations (translated from Norwegian into English by AOH) from the interviews in order to ensure the anonymity of the participants.

## Results

### Participants

A total of 7 physiotherapists participated in this study, 1 man and 6 women between the age of 38 and 71 (mean 39) years. They had worked in rehabilitation between 8 and 37 (median 15) years. The participants were physiotherapists with advanced expertise in rehabilitation. Education levels varied from bachelor’s and master’s to doctoral degrees in physiotherapy; however, all had completed courses and further education within the field to be titled specialist physiotherapist. In total, 5 physiotherapists had been certified to either level 1 or level 2 of the supplier’s required training, which involves using the robot with more advanced settings. Each had between 8 and 37 years of clinical experience, with several serving in senior or leadership positions within specialized rehabilitation centers. This level of expertise provided an in-depth understanding of the factors influencing user acceptance of technology in a clinical setting. The level of experience using the exoskeleton varied from weekly use to almost no use in the past year. All the participants had started using the exoskeleton in the hospital’s implementation project 3 years before data collection.

### Results From Content Analysis

#### Overview

Table 3 presents the findings based on the main categories of the UTAUT framework. Findings related to subcategories are described in the text, illustrated with citations representing all informants.

**Table 3:** Summary of the study findings. Main categories and subcategories aligned with the constructs of the unified theory of acceptance and use of technology framework.

Main category	Subcategories
Performance expectancy	Perceived benefits of using the robot Perceived disadvantages of using the robot
Effort expectancy	Robot design and functionality Customization and personalization of the robot
Social influence	Managers’ influence on use behavior Peers’ influence on use behavior
Facilitating conditions	Available resources Training and expertise
Behavioral intention	Intentions to adopt the robot Intentions not to adopt the robot

#### Performance Expectancy

##### Perceived Benefits of Using the Robot

The exoskeleton was described as a good supplement to existing walking training and its usefulness lay in the possibilities it added. All the participants described the benefits of using the robot, such as the ability for a patient to stand up, facilitating an increased number of steps, or symmetrical loading of the body in treatment. The usefulness for patients varied depending on the individual’s functional abilities and diagnosis. For example, the exoskeleton offered new opportunities, especially for patients with complete spinal cord injuries:

If you have a complete spinal cord injury, you don’t have the ability to walk. For these patients, it’s been a great opportunity to be able to stand up straight and walk. Using the exoskeleton may also facilitate secondary treatment benefits such has cardiovascular effects which are also achieved by mobilization.

Furthermore, the increased number of steps was considered particularly beneficial in spinal cord injury training that focused on neuroplasticity:

The patients can focus more on performing an increased number of steps. It [the robot] is stable, I control the balance. The patients can work on weight transfer. The steps come on their own or on impulse from the patients. So, they get a lot more repetitions by using the robot, because it’s automatically possible to do more repetitions. That’s clearly an advantage in using a walking robot.

Participants who described benefits, such as symmetrical loading, mostly worked with patients who had experienced a stroke. One of the benefits they described was related to the possibility of “disconnecting” one of the exoskeleton’s legs to allow the good leg to move freely. Other benefits described were related to the fact that the exoskeleton stabilized the patient quite well, and this simplified the work of the physiotherapists and freed up their hands so they could focus on other aspects of the treatment.

Furthermore, several participants stated that it was an advantage that the speed of the exoskeleton was slow, which made it easier to focus on the execution of movements and thus increased the quality of the treatment. It was also considered an advantage that the robot did not let patients compensate with a shorter stance phase on the affected side; rather, it supported the full weight transfer of their whole body.

Several participants also talked about the benefit of being able to help patients feel safer by creating safe and stable frameworks, which meant that patients dared to challenge themselves more. Furthermore, the experience of standing upright also seemed very motivating for the patients and, in that way, contributed to something beyond traditional walking training for certain patient groups.

##### Perceived Disadvantages of Using the Robot

Using the exoskeleton was quite time consuming, which was described by all the participants as a significant disadvantage. The initial treatment was perceived as particularly time consuming due to all the preparations, adjustments, and measurements that needed to be made. When the exoskeleton was used on different patients, the disadvantage related to time consumption was amplified. The time required for adjustments both before and after the training session made the disadvantages outweigh the benefits, as the time that could have been saved in terms of quantity and quality of treatment was consumed by preparation and adjustments. Some participants described it as taking time away from effective treatment time. However, participants reported reduced time consumption during subsequent training sessions in comparison to the first one.

Several participants mentioned that the exoskeleton could negatively affect patients’ self-effort and expectations. For example, some participants described how the patient was confined within the “skeleton” of the exoskeleton, which made it difficult to assess the patient’s own effort. This resulted in uncertainty about the effectiveness of treatment for several patients. Some participants explained this by describing how the exoskeleton could function as a crutch. One informant stated the following:

When they [patients] started getting better, it became apparent that they relied more on the robot’s support to a greater extent than I had hoped...because something unconscious happens when you are so encapsulated that you automatically rely a bit more on the robot, and then it becomes difficult to challenge yourselves enough.

Similarly, some physiotherapists described a downside to the benefits of the technology that dealt with the challenge of managing patient expectations, for example, for patients with spinal cord injury who could have high hopes or hopes of regaining function beyond what was realistic. Finally, it was mentioned that sometimes, patients used the robot for such a brief period that it was not effectively used. As a result, the physiotherapists did not experience any of the mentioned benefits of its use.

#### Effort Expectancy

##### Robot Design and Functionality

Certain physiotherapists said that the exoskeleton was large and heavy to handle. One suggested that it could have been made from completely different materials. The size and weight of the exoskeleton were perceived as particularly challenging when mobilizing patients from a chair and onto the exoskeleton and when supporting activity during walking training itself. Due to its weight, moving the equipment to other locations was out of the question. Few participants said that a ceiling rail had been installed to assist with this problem.

Most of the participants perceived that it was easy to learn how to use the exoskeleton. One described it as follows:

It [the robot] is easy to use in the sense that it can be learned by reading an instruction manual.

At the same time, most of the participants thought the robot was very comprehensive and time consuming to use. This was partly explained by the fact that the physiotherapists had to conduct manual adjustments and programming of the robotics before using it. Some said that they had some kind of checklist they went through, which made it easy to get the basic settings right. Furthermore, many experienced the user interface on the exoskeleton as “old-fashioned” and not very user-friendly. It was pointed out that the menu system had many setting options, but there were few buttons for selecting settings. Finding the setting they wanted to use required several operations, which was exacerbated when using the technology’s most advanced settings. As a result, most of the participants did not use the advanced settings. One of the informants summarized this as follows:

It’s not user-friendly at all, I feel, because there are far too many possibilities on too few buttons, I think, so you have to scroll forever to find what you’re looking for, even then you have to kind of learn by heart which functions are under which in the menu—so in relation to using a smartphone, it was in a way so far back in relation to those systems, you felt in a way that you were using an old personal computer.

Some participants also mentioned that the technology felt “unnecessarily complex.” One of the informants described it as follows:

We have a lot of technical aids here in the facility, but the Ekso is clearly the most comprehensive of them all.

##### Customization and Personalization of the Robot

Some participants experienced that the robot device had physical limitations, which meant that some patients were too tall, too short, too wide, or too narrow to fit it. There was no solution to this, and several participants described this as a major limitation. One of the informants said the following:

It was a big limitation, especially on this issue of hip width, we were often adamant about it. There were several patients where we thought it would fit, but then they were too wide over their bum, it also happened that they were too small for it to fit. Or too tall or short.

The physiotherapists described that if the exoskeleton was used frequently with different patients, there was no quick way to use the settings that had been used on the patient during a previous treatment. All settings had to be replotted. Challenges surrounding the need for manual adjustments to the device, such as adaptation and adjustment of the ankles, knees, and hips, were described by several of the participants. Some expressed that this was a matter of training and that one got better at adjustments when one had done it enough times. Some of the participants also described a fear of tightening the straps too tightly or making incorrect settings that could make it uncomfortable or create fear in the patients.

The participants were asked what could make the robot easier to use. Several said that ease of use for them in relation to the robot concerned whether it was quick to prepare for use, that it required few work operations, and that the user interface was clear and intuitive.

#### Social Influence

##### Managers’ Influence on Use Behavior

The participants experienced that the management’s commitment to and willingness in the implementation of the exoskeleton influenced their use of it to a large extent. The management’s wish and plans to adopt the exoskeleton were communicated clearly, and this was followed by resources and goodwill to facilitate its implementation. The participants said that the management encouraged the inclusion of patients whose treatment could fit the use of the exoskeleton. These calls were followed up by the physiotherapists in many different treatment settings, which led to frequent use of the exoskeleton.

Some participants suggested that the hospital management’s aim and vision of being at the forefront of new technology and innovation played a role in why they chose to adopt the technology. They had also experienced that when other, and sometimes conflicting, innovation projects related to gait training came along, the enthusiasm and follow-up had somewhat decreased. One of the informants said the following:

We’re supposed to do a lot of cool projects, but there’s perhaps too little holistic thinking around walk training.

Some participants also proposed that when the need for certain technology has not come “from the therapists,” it may be difficult to follow calls that come from the management. One participant said the following:

You could say that the desire to use [the exoskeleton] did not come from the therapists. It has come from the top management, and it’s a slightly unnatural way to approach a product—a bit like: Now you have it, so now you have to figure it out.

Because the implementation of the exoskeleton had not been triggered by the needs of the therapists, some participants felt it was a form of forced use.

##### Peers’ Influence on Use Behavior

Most participants said that the attitudes of their colleagues toward the exoskeleton did not matter much in relation to their own choice to use it. One stated that they had not experienced any negative attitudes toward using such a robot. Some participants shared that one could be influenced to use it if colleagues brought it up in plenary meetings, that is, asking for possible candidates for using the exoskeleton. Other participants proposed that professional discussions about the exoskeleton influenced their choice to adopt the technology. One participant said the following:

We had colleagues who were quite positive about it. At the same time, people wondered a little whether it would work for the patients we were going to use it for.

#### Facilitating Conditions

##### Available Resources

Many participants described organization-related challenges in using the exoskeleton. Several mentioned that its use was perceived as resource intensive, as it required 2 people to be involved in the treatment and its use and thus needed to be coordinated with other staff. One explained it as follows:

It’s a problem in the healthcare system in general that we don’t have an abundance of staff. So, it became quite cumbersome to arrange it, to schedule the use of the exoskeleton, and to find someone to assist during the same time slot and manage the logistics so that it could work.

Due to this, the use of the robot was perceived as vulnerable in case one of the therapists was absent from work. One participant stated the following:

I think the resources are not there in terms of personnel.

Several participants mentioned that the use of the exoskeleton worked better during certain periods when they had a porter available to prepare the equipment before use. At the same time, some noted that if staff had to be reassigned to other tasks, they had to cancel the exoskeleton treatment due to staffing needs. Furthermore, most participants mentioned that when they needed to use the exoskeleton, they sometimes had to deprioritize something else that was already scheduled. This affected not only their own time allocation but also that of the other therapists who had to participate. Because the use of the exoskeleton was so time consuming, several of the participants who worked with patients with spinal cord injuries perceived that the exoskeleton was not suitable for the interdisciplinary program that patients went through during their stay.

The location of the exoskeleton in the gym was considered appropriate and well suited to its use. This was partly explained by the fact that it felt safe that there were more people available in the room. In addition, a rail with suspension from the ceiling had been added, which reduced the need for personnel. Other participants felt that the location in the gym made the exoskeleton less relevant for some patients because some patients had challenges that made it difficult if there were too many other people in the same room.

Several of the physiotherapists said that a large part of the resource needs would be solved if it were organized as a “lab” and that this could solve several of the challenges related to time and personnel resources. One participant said the following:

There should be a separate laboratory where one uses such technology, where there are staff who do this job. If we had such lab, [the exoskeleton] would probably have been used more because people would then be able to try it out, while they received other training and treatment, which they needed in addition. That more patients could be offered to try the Ekso.

Regarding technology support, participants knew whom to ask if they were unsure about something regarding the device. They had superusers for support if needed.

##### Training and Expertise

All the participants perceived the training on using the exoskeleton as very useful. Some said that the training provided the skills needed to put it into use, and several described the training as having a good balance between theoretical and practical exercises. Some of the participants stated that the training was “very extensive,” especially in order to be certified to level 2, which was required for being responsible for treatment with the use of the robot. The extensive part of the treatment was described as time consuming due to the need for an instructor from the robot supplier to be there for 1 week, in addition to several hours required afterward to be certified. Others described it as extensive due to the fact that their daily schedule was very tight, and they had to be replaced by other therapists.

The participants were satisfied with the training they had received, but, at the same time, they asserted that one had to practice a lot to become really good at using the exoskeleton and its advanced settings. One participant proposed the following:

Like with all new things, time must be set aside for someone to become very good at it.

It was suggested by several participants that it could be an advantage to organize the use of the robot within a laboratory and engage certain physiotherapists to gain and maintain good competence in using the robot.

#### Behavioral Intention

##### Intentions to Not Adopt the Robot

The physiotherapists’ biggest reasons for choosing not to use the exoskeleton referred to the time consumption and disadvantages of use, as described in the Performance Expectancy section, and to overly extensive planning, as described in the Facilitating Conditions section. Other reasons were their own professional judgment and the lack of evidence-based knowledge about the effects of using such a robot. One of the informants described some of this as follows:

For me, it’s important that there’s a professional foundation. It’s not enough that it’s technological and cool, or that we have to show off. The most important thing for me is what benefits the patient or not.

Furthermore, several of the physiotherapists described how the acquisition of a walking robot was not triggered by a need from the therapists in the clinic. Some also found it difficult to know when and what to use it for. One of the informants said the following:

It must be said that this is also where a bit of our opposition lies—without having any good professional arguments for using it—the managers overlook our professional judgements on what is reasonable treatment and use of resources.

All the participants expressed that the possible benefits that could be achieved in one treatment session using the exoskeleton were eaten up by the time spent on rigging it and the several operations related to its use. Several said that the need for 2 physiotherapists when using it was one big reason for choosing not to use the exoskeleton. It could be challenging to coordinate with another physiotherapist’s timetable, or this could result in a second physiotherapist prioritizing to assist before conducting other valuable treatments. Both the planning and the logistics related to the exoskeleton treatment session were so time consuming and difficult to achieve that it could be perceived as easier to choose not to use the exoskeleton.

##### Intentions to Adopt the Robot

What the physiotherapists described as the biggest reason for choosing to use the exoskeleton was that it offered advantages for some patients, such as new opportunities to be able to stand up straight and walk. These benefits were related to those patients who had some gait function but could not yet practice traditional walking training. Others chose to use the exoskeleton to get people with spinal cord injuries, who otherwise would not be able to do so, to stand. The exoskeleton could provide a safe environment for insecure patients, and it could be used with patients who were motivated by using new technology. Furthermore, several participants described certain criteria for using the exoskeleton, such as consulting the responsible therapist and investigating whether there were contraindications due to specific medical conditions and whether it fitted the patient’s physical goals.

Some participants said that the decision to use the exoskeleton initially was made because it was necessary to log hours for certification during the implementation project. Several of the participants who described this used it most frequently in that period. Other participants described that a priority on new technology being used was the reason for choosing to use it.

When asked what would be needed for the technology to be used more frequently in the future, the participants described how more resources and time were needed. Several said that it must “require less energy” for it to be used more frequently. Among other things, some participants pointed out that it will be important to start treatment early as a supplement in order to choose to use it. In addition, it must fit in with the way they work and their needs. For example, several of the participants highlighted the need to gather expertise and resources in a “lab.” Here, the exoskeleton could be organized in such a way that one could book an appointment with one’s patient, and it would not come at the expense of other treatment for one’s other patients.

Several participants called for more evidence-based knowledge about the treatment effects before one could professionally defend using the time and resources needed to use the exoskeleton:

It becomes a dilemma in relation to what to invest in using it, when you don’t know the actual effects. What we’ve seen from articles and such has not been very encouraging.

Other improvement points that the participants described dealt with the development of the exoskeleton technology itself. Some argued that the weight and size of the device should be reduced to expand the range of use and that the control panel should be more intuitive and user-friendly.

## Discussion

### Principal Findings

#### Overview

To the best of our knowledge, this study is among the first to investigate the use of a lower limb exoskeleton in specialized neurological rehabilitation using the UTAUT framework, based on the experiences of expert physiotherapists. The physiotherapists predominantly perceived the disadvantages of using the exoskeleton as outweighing the benefits. The use of the exoskeleton was significantly influenced by the perceived benefits for patients, its time and personnel demands, the adequacy of training and support, clinical evidence, and accessibility. A cost-benefit perspective on its use and perceived advantages was decisive in determining how frequently they chose to use the exoskeleton in clinical practice.

The exoskeleton was perceived as beneficial for rehabilitating patients with limited mobility, especially those with spinal cord injuries, by enabling them to stand and walk. It increased the number of steps in training, enhanced neuroplasticity, and promoted symmetrical weight distribution. Its stability allowed physiotherapists to focus on other treatment aspects, and patients felt safer and more motivated.

However, significant drawbacks included the time-consuming setup, especially during initial sessions, which reduced treatment efficiency. The exoskeleton’s bulk and outdated user interface also posed challenges, making it cumbersome to handle and adjust. Physiotherapists expressed concerns that patients might rely too heavily on the device, undermining their own efforts. In addition, staffing constraints further limited its use, as 2 physiotherapists were required per session.

The findings are further discussed in accordance with the main categories of the UTAUT framework.

#### Performance Expectancy

The findings showed that the physiotherapists’ perceived usefulness (performance expectancy) of the exoskeleton was primarily related to the possibilities and benefits it offered in treating their patients. They highlighted advantages, such as enabling patients to stand up straight, increasing the number of steps taken, and promoting symmetrical body loading. The experiences described by our participants regarding physical and diagnosis-dependent benefits align with previous studies by Vaughan-Graham et al [3] and Mortenson et al [13].

However, despite the aforementioned advantages, most physiotherapists identified the time required to prepare the exoskeleton for initial treatment as a significant disadvantage, which is consistent with findings from Read et al [2]. Additional perceived disadvantages revolved around the narrow scope of the exoskeleton’s utility. The technology was perceived as effective only within a specific clinic area, depending on the diagnosis, and for a limited duration during rehabilitation. As patients’ functions significantly improved, conventional gait training was preferred. Conversely, if patients’ conditions were poor, adaptations were time consuming and deemed inefficient. This may be related to what several physiotherapists described as a “free ride,” where patients were essentially passive, without significant therapeutic effect. This aligns with Turchetti et al [24], who identified this potential downside of robot technology in rehabilitation. One possible explanation for this issue is the difficulty physiotherapists face in assessing patients’ exertion and contribution when they are confined within the exoskeleton. Another explanation could be related to the level at which physiotherapists use the exoskeleton. Few reported using it at a “high level” fully exploiting all the device’s functionalities.

The physiotherapists predominantly perceived the disadvantages of using the exoskeleton as outweighing the benefits. This cost-benefit perspective is elucidated by Mortenson et al [13], who raised questions regarding the exoskeleton’s efficacy and cost-effectiveness relative to other approaches. However, our findings are consistent with those of Liu et al [19], indicating that physiotherapists are likely to adopt new technology if they perceive it as beneficial for their patient-related work. For example, our participants found that the benefit of using the exoskeleton for certain patients was a key motivation for their intention to use it. Thus, our findings are in line with the UTAUT framework [17], showing that performance expectancy, herein perceived usefulness (or lack thereof), is the strongest predictor of user acceptance and intention to use technology.

Similar to participants in other studies [3,11,25], the physiotherapists expressed disappointment regarding their expectations of the potential benefits of using the technology. This may suggest a discrepancy between user needs and the acquired product, a known issue with acquisitions of commercial off-the-shelf solutions, as described by Turchetti et al [24]. Challenges associated with such acquisitions include products not being developed to address the needs they are meant to solve. Physiotherapists find it difficult to understand which requirement the exoskeleton is intended to fulfill. This can be explained, on the one hand, by the nature of the product but also by the absence of user engagement during the implementation process, a phenomenon commonly referred to as the “top-down” approach in the literature [26,27].

#### Effort Expectancy

The physiotherapists’ effort expectancy was related to the design, functionality, customization, and personalization of the exoskeleton for each patient. For example, the exoskeleton was challenging to use due to its size and weight, and its outdated control panel (user interface) with limited options made adjustments unnecessarily complex. However, despite its perceived lack of user-friendliness, most participants found learning how to use it easy.

Effort expectancy in terms of usability is expected to significantly influence the degree of use during the initial phase of implementation when users have not yet learned how to use the technology [17]. Previous studies have demonstrated that the impact on the intention to use decreases the longer the technology has been in use regardless of how difficult it is to learn [17,19]. On the one hand, the findings of this study align with this notion, as the physiotherapists have been using the technology for >3 years, and perceived usability had less influence on their intention to use it. Some participants expressed that “it was a matter of training” and that proficiency improved with practice. On the other hand, several physiotherapists used the technology sparingly over these 3 years, suggesting that perceived usability might be more significant than the results indicate. A possible explanation is that all physiotherapists in this study found the gait robot easy to learn initially.

In this study, perceived usability has been shown to have little influence on physiotherapists’ intention to use the gait robot, consistent with the findings of Liu et al [19]. For the exoskeleton to be perceived as more user-friendly, the participants suggested that such technology should be quick to prepare for use, requiring few operational steps, and having an interface that is clear and intuitive.

#### Social Influence

According to Venkatesh et al [17], social influence from others may directly influence behavioral intention. In addition, a moderating factor on social influence is the voluntariness of use. The physiotherapists in this study perceived they were influenced by their management, particularly at the onset of the implementation project when it was necessary to log hours to achieve certification. Some also indicated that the exoskeleton was a “top-down” investment not triggered by the physiotherapists’ needs. In contrast to Venkatesh et al [17], our findings indicate minimal influence from colleagues on their behavioral intention.

Upon reviewing the literature on the use of rehabilitation robots, no other studies were found that described findings related to social influence.

#### Facilitating Conditions

According to Venkatesh et al [17], facilitating conditions have a direct impact on user acceptance. This was supported in our findings, as physiotherapists described shortcomings related to available resources, organization, training, and competence related to using the exoskeleton.

The findings of our study largely revolved around the disadvantages that physiotherapists experienced related to organization and resources. This affected physiotherapists’ behavioral intention in such a way that they demanded more resources in terms of personnel and time to further use the exoskeleton. This aligns with the challenges described by Read et al [2], Mortenson et al [13], and Reicherzer et al [16]. Furthermore, our findings indicated that facilitating conditions, such as a lack of organization and resources, affected behavioral intention to a greater extent than their use behavior. This is in accordance with the findings of Liu et al [19] who found that physiotherapists who perceive good utility value use the technology regardless but more frequently if facilitating conditions were in place. Liu et al [19] referred to how facilitating conditions are the strongest factor influencing physiotherapists’ use of new technology in the rehabilitation setting.

The finding that physiotherapists experience challenges related to planning and providing treatment on a hectic weekday aligns with the experiences of physiotherapists in the study by Mortenson et al [13]. Physiotherapists in our study emphasized the significant resource demands associated with coordinating appointments involving multiple physiotherapists for treatment, particularly in terms of personnel allocation. This was also found in the study by Mortenson et al [13]. Multiple physiotherapists described untapped potential in the current implementation of the technology, a recognized concern based on research in health technology and implementation [26,28].

It is important to note that the implementation of RAGT requires a large investment in training and equipment for most services.

#### Behavioral Intention

According to Venkatesh et al [17], behavioral intention captures the individual’s motivation and intention to use the technology. They also described behavioral intention as a key predictor of actual use behavior when interacting with technology.

Physiotherapists in our study cited the exoskeleton’s potential to benefit patients, particularly those with limited function or spinal cord injuries, as a key motivation for adoption. The technology provides new opportunities for patients to stand and walk and fosters a safe, motivating environment. In addition, participants identified specific criteria for use, including consultation with responsible therapists and consideration of patient goals. However, the lack of available resources within the organization, including time and personnel, emerged as a central barrier to adoption. Physiotherapists expressed concerns about the time-consuming nature of using the exoskeleton and the logistical challenges associated with coordinating sessions. In addition, uncertainty surrounding the technology’s effectiveness and the need for evidence-based knowledge were highlighted as deterrents to adoption.

Our findings align with previous research by Liu et al [19], which identified performance expectancy and intention to use as key predictors for actual use. Similarly, Heinemann et al [11] and Mortenson et al [13] emphasized the importance of expanding the scope of use scenarios to promote future adoption. This aligns well with our findings in that physiotherapists experienced that for particular patients and particular rehabilitation phases, the robot simply could not be used. This was related to patients who had not obtained enough function to take advantage of the robot yet, but the time frame, between being ready to use the robot and when they were more likely to walk without it, was fairly short, limiting the “window of opportunity” for those particular patients and rehabilitation phases.

Our study uniquely highlights the potential barrier posed by the lack of available resources within the organization, a factor not extensively explored in previous literature. This underscores the need for a comprehensive examination of user acceptance among physiotherapists to guide the implementation of robot-assisted training in clinical practice, as highlighted by Venkatesh et al [17] and our own study.

### Strengths and Limitations of This Study

The data and findings in our study are unique, as we applied the validated UTAUT framework in exploring physiotherapists’ acceptance of a robotic lower limb exoskeleton in specialized neurological rehabilitation. To increase the validity and reliability of the findings, the main categories of the deductive analysis were based on the dimensions of the UTAUT framework. Knowing that using a deductive or directed content analysis approach may bias the identification of other possible main categories or subcategories in the text [29], the authors remained open minded during the analysis to allow for the emergence of additional themes or subthemes from the data.

The trustworthiness of the study was ensured using the criteria of credibility, dependability, and transferability [30]. Credibility can be understood as maintaining a careful focus on the project. This criterion was met by choosing participants who were most relevant to the aim of this study. Dependability was ensured through rigorous and well-documented data collection techniques and procedures. The interview guide was pretested, and the same questions were used for all the participants. Moreover, all interviews were conducted by the first author, who is a physiotherapist with extensive expertise in the use of technology in health care. The other authors have extensive expertise in specialized rehabilitation, pedagogics, and qualitative research, which influenced the study design, data collection, and data interpretation throughout the research process.

Although the generalization of findings is not a goal of qualitative research, qualitative researchers strive for the transferability of findings. To address this, we provided a thorough description of the context, participants, and research process. Furthermore, we attempted to present the findings in as much detail as possible while ensuring the deidentification of the informants. Thus, we argue that the qualitative part of the study meets the criteria for trustworthiness.

There are some limitations to this study. Due to the scarcity of physiotherapists certified in the use of the lower limb robotic exoskeleton in a specialized rehabilitation unit, the eligible sample was limited. Even so, despite the small sample size, it can be argued that information power was achieved, as the cohort of expert physiotherapists provided substantial insights relevant to the actual study [31]. The findings reflect the perspectives of physiotherapists at a specialized rehabilitation hospital in Norway. However, we think the findings may be transferable to comparable contexts.

### Conclusions

Our findings showed that the participants’ intention to use and their actual use of a lower limb exoskeleton in a specialized rehabilitation setting were influenced by perceived benefits (performance expectancy) for patients; challenges, such as usability issues and the time required for adjustment to each patient (effort expectancy); the lack of personnel resources to facilitate its use (facilitating conditions); and deficient evidence-based knowledge about its treatment effects. A cost-benefit perspective was adopted where the perceived advantages were a decisive factor in determining how frequently they chose to use the exoskeleton. The expert physiotherapists predominantly perceived the disadvantages of using the exoskeleton as outweighing the benefits.

This study advances understanding of the factors affecting the user acceptance of lower limb RAGT in specialized rehabilitation from the perspective of physiotherapists. Although the findings are limited by the sample size and the exploratory nature of the study, they provide directions for possible decisive factors for the acceptance and integration of lower limb exoskeletons in specialized rehabilitation. With a growing demand for technology in rehabilitation, our insights on acceptance and integration of the lower limb exoskeleton may inform others who are considering or in the process of integrating exoskeletons into clinical practice. The findings may inform exoskeleton engineering, researchers, and service providers in Norway and beyond.

Further research should focus on gathering evidence on the rehabilitation effectiveness and cost-effectiveness of lower limb exoskeletons. Such evidence can justify their integration into specialized rehabilitation by demonstrating clinical benefits and economic viability.

## Abbreviations

COREQ: Consolidated Criteria for Reporting Qualitative Research
RAGT: robot-assisted gait training
UTAUT: unified theory of acceptance and use of technology
